# Transglutaminase 2 Contributes to Apoptosis Induction in Jurkat T Cells by Modulating Ca^2+^ Homeostasis via Cross-Linking RAP1GDS1

**DOI:** 10.1371/journal.pone.0081516

**Published:** 2013-12-11

**Authors:** Yu-Fan Hsieh, Guang-Yaw Liu, Yi-Ju Lee, Jiann-Jou Yang, Katalin Sándor, Zsolt Sarang, Angela Bononi, Paolo Pinton, László Tretter, Zsuzsa Szondy, Gregory J. Tsay

**Affiliations:** 1 Institute of Microbiology and Immunology, Chung Shan Medical University, Taichung, Taiwan; 2 Department of Biomedical Sciences, Chung Shan Medical University, Taichung, Taiwan; 3 Department of Biochemistry and Molecular Biology, Research Center of Molecular Medicine, University of Debrecen, Debrecen, Hungary; 4 Department of Experimental and Diagnostic Medicine, Section of General Pathology, Interdisciplinary Center for the Study of Inflammation (ICSI), Laboratory for Technologies of Advanced Therapies (LTTA), University of Ferrara, Ferrara, Italy; 5 Department of Medical Biochemistry, Semmelweis University, Neurobiochemical Group of Hungarian Academy of Sciences, Budapest, Hungary; 6 Department of Internal Medicine, Chung Shan Medical University Hospital, Taichung, Taiwan; Karolinska Institutet, Sweden

## Abstract

**Background:**

Transglutaminase 2 (TG2) is a protein cross-linking enzyme known to be associated with the *in vivo* apoptosis program of T cells. However, its role in the T cell apoptosis program was not investigated yet.

**Results:**

Here we report that timed overexpression of both the wild type (wt) and the cross-linking mutant of TG2 induced apoptosis in Jurkat T cells, the wt being more effective. Part of TG2 colocalised with mitochondria. WtTG2-induced apoptosis was characterized by enhanced mitochondrial Ca^2+^ uptake. Ca^2+^-activated wtTG2 cross-linked RAP1, GTP-GDP dissociation stimulator 1, an unusual guanine exchange factor acting on various small GTPases, to induce a yet uncharacterized signaling pathway that was able to promote the Ca^2+^ release from the endoplasmic reticulum via both Ins_3_P and ryanodine sensitive receptors leading to a consequently enhanced mitochondrial Ca^2+^uptake.

**Conclusions:**

Our data indicate that TG2 might act as a Ca^2+^ sensor to amplify endoplasmic reticulum-derived Ca^2+^ signals to enhance mitochondria Ca^2+^ uptake. Since enhanced mitochondrial Ca^2+^ levels were previously shown to sensitize mitochondria for various apoptotic signals, our data demonstrate a novel mechanism through which TG2 can contribute to the induction of apoptosis in certain cell types. Since, as compared to knock out cells, physiological levels of TG2 affected Ca^2+^ signals in mouse embryonic fibroblasts similar to Jurkat cells, our data might indicate a more general role of TG2 in the regulation of mitochondrial Ca^2+^ homeostasis.

## Introduction

Transglutaminases are a family of thiol- and Ca^2+^-dependent acyl transferases that catalyze the formation of a covalent bond between the γ-carboxamide groups of peptide-bound glutamine residues and various primary amines including the ε–amino group of lysine in certain proteins [1]. The reaction results in post-translational modification of proteins by establishing ε–(γ–glutamyl)lysine cross-linkages and/or covalent incorporation of polyamines and histamine into proteins. Transglutaminase 2 (TG2) is a very unique member of the transglutaminase family, because besides being a transglutaminase it also possesses GTPase, protein disulphide isomerase and protein kinase enzymatic activities [2]. In addition, TG2 can also function in various biological settings as a protein/protein interaction partner. For example, the protein also possesses a BH3 domain, thus it is believed to contribute to the initiation of apoptosis by interacting with members of the Bcl-2 family [3].

Apoptosis, the dominant cell death form of mammalians, is characterized morphologically by membrane blebbing, chromatin condensation, DNA fragmentation, and formation of apoptotic bodies, which are engulfed by neighboring cells [4]. Studies at the molecular mechanism have suggested that mitochondria play the central role in the initiation of the intrinsic pathway of apoptosis by responding to numerous apoptosis-inducing signals with release of various pro-apoptotic factors [5]. Both mitochondria and endoplasmic reticulum (ER) are stores for intracellular calcium (Ca^2+^), and are closely associated via 5 to 20% of the mitochondrial membrane surface being attached to ER membrane domains named mitochondria-associated membranes (MAMs) [6]. Apoptosis-related studies have demonstrated that fine tuning of the mitochondrial Ca^2+^ homeostasis by pro- and anti-apoptotic proteins plays a determinant role in the regulation of apoptosis [7], and increased mitochondrial Ca^2+^ uptake facilitates the initiation of the apoptotic process [8], [9]. The source of Ca^2+^ is the ER, which, upon the administration of the apoptosis-inducing stimuli, releases it directly into the mitochondria via the inositol-1,4,5-trisphosphate receptor (InsP_3_R) type III located in the MAMs [10], [11].

TG2 expression has been known for a long time to be associated with the *in vivo* apoptosis program [12]. While in certain cancer cell types overexpression of TG2 increases survival [13], in many other cells, including T cells, the protein seems to act as a pro-apoptotic molecule. TG2 is not expressed by living thymocytes. However, the protein is strongly induced in thymocytes following exposure to various apoptotic signals *in vivo*, and it appears in the initiation phase of apoptosis [14]. Isolated thymocytes exposed to pro-apoptotic signals also die, but do not upregulate TG2 implying that the apoptosis initiation does not require TG2. Upregulation of TG2 *in vivo* is mediated by co-signals provided by the surrounding engulfing macrophages [15]. In addition to dying thymocytes, TG2 also appears in the dying T lymphocytes of HIV-infected individuals [16]. While TG2 was shown to promote apoptosis by expressing its BH3 domain [3], by cross-linking the retinoblastoma protein [17] as well as by phosphorylating P53 [18], so far the role of TG2 in the T cell apoptosis program has not yet been investigated in details. Here we report that timed overexpression of both the wild type (wt) and the cross-linking mutant of TG2 (TG2X) induced apoptosis in Jurkat T cells, the wt being more effective. Part of TG2 colocalised with mitochondria containing increased amount of calcium. Overexpressed wtTG2 cross-linked RAP1, GTP-GDP dissociation stimulator 1 (RAP1GDS1), an unusual guanine exchange factor acting on various small GTPases [19], which appeared in the ER to induce a yet uncharacterized signaling pathway that was able to promote the Ca^2+^ release from the ER via both Ins_3_P and ryanodine sensitive receptors leading to an enhanced mitochondrial Ca^2+^ uptake. Our data indicate that TG2 might act as a Ca^2+^ sensor in the mitochondria to amplify ER-derived Ca^2+^ signals, and demonstrate a novel mechanism through which TG2 can contribute to the induction of apoptosis in T cells.

## Results

### Generation of Jurkat T cells with inducible expression of TG2

**Figure 1:**
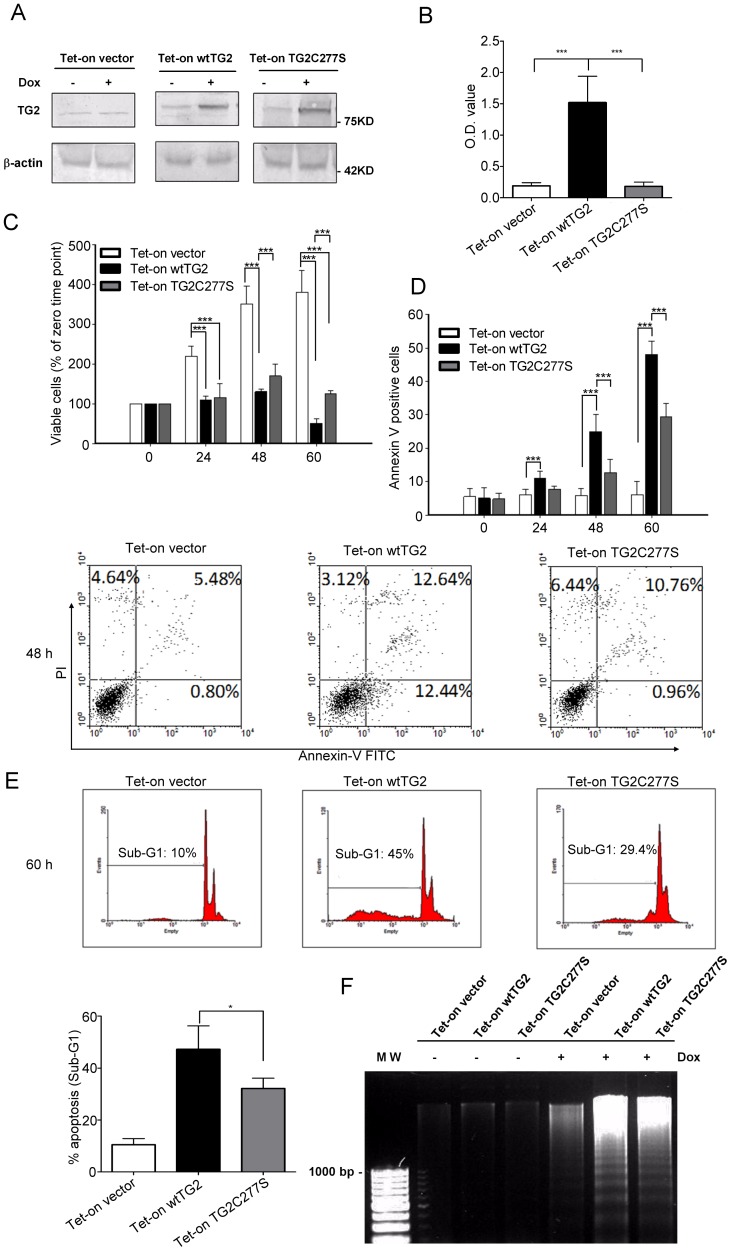
Timed overexpression of wtTG2 or of its cross-linking mutant induces apoptosis in Jurkat T cells. (A) Tet-on vector, Tet-on wtTG2 and Tet-on TG2C277S cells were treated with 50 µM doxycycline (Dox). The expression level of TG2 protein was detected by Western blot analysis after 18 h of Dox treatment. β-actin was used as a loading control. (B) The transglutaminase enzyme activity in the various types of Jurkat cells was determined after 6 h of Dox treatment. (C) Induced overexpression of wtTG2 or TG2C277S decreased the cell viability in a time dependent manner. Number of viable cells was determined at the indicated time points following Dox treatment. Significantly different from the viable cell number of the indicated cell line at the same time point (***P<0.001). (D) Induced overexpression of wtTG2 or TG2C277S increased the percentage of Annexin V positive cells. Number of Annexin V and/or propidium iodide positive cells was determined at the indicated time points following Dox treatment. Significantly different from the % of Annexin V positive cells of the indicated cell line at the same time point (**P<0.01; ***P<0.001). Flow cytometric data demonstrate results detected after 48 h of Dox treatment. (E) DNA histogram of propidium iodide stained Tet-on vector, Tet-on wtTG2 and Tet-on TG2C277S cells treated with Dox for 60 hours. Cells exhibiting sub-G1 levels of DNA were considered apoptotic and their amount was calculated as percentage of the total cell number. *Data represent at least three independent experiments and are shown as mean ± SD (*P*<0.05). (F) Electrophoretic analysis of internucleosomal DNA fragmentation in Tet-on vector, Tet-on wtTG2 and Tet-on TG2C277S cells treated with or without Dox (50 µM) for 60 hours. MW, standard. The results are representative of one of three independent experiments.

In order to investigative the effect of TG2 overexpression in T cells, Jurkat cells were transfected with the wild-type (wtTG2) and a cross-linking mutant of TG2 created by replacement of the catalytic Cys^277^ by Ser [20]. The wtTG2 or mutant TG2-overexpression was driven by a Jurkat (JK)-tetracycline (Tet)-on inducible promoter. Western blot (Figure 1A) analysis showed a similar degree of increase in protein expression of TG2 in JK-Tet-On wtTG2 and mutant TG2 cells upon 50 µM Doxycycline (Dox) treatment. However as expected, the TG2 transamidase activity increased notably only in in JK-Tet-On wtTG2 cells (Figure 1B).

### Overexpression of both wild type and cross-linking mutant of TG2 induces apoptosis in Jurkat T cells

Following exposure to Dox, there was a marked decrease in the doubling time of Jurkat T cells expressing either the wt or the cross-linking mutant TG2, as compared to the vector expressing line. In accordance, a significantly lower viable cell number could be detected at 24, 48 and 60 h by using the MTT assay (Figure 1C). The decreased amount of viable cells was a consequence of cell death induction by overexpressed TG2 in Jurkat T cells, as the percentage of Annexin V positive cells was significantly increased with time (Figure 1D). The death induced seem to be apoptosis, as DNA histograms demonstrated increased number of cells expressing degraded DNA (Figure 1E). In addition, we could detect DNA ladder formation characteristic for apoptotic cells (Figure 1F) [21]. However, the rate of apoptosis was faster in Jurkat T cells over-expressing wtTG2 than its cross-linking mutant indicating that both the cross-linking and the other biological activities of this multifunctional protein contribute to the apoptosis induction in these cells.

### Overexpressed TG2 appears in the mitochondria and enhances intra-mitochondrial Ca^2+^ concentration

**Figure 2:**
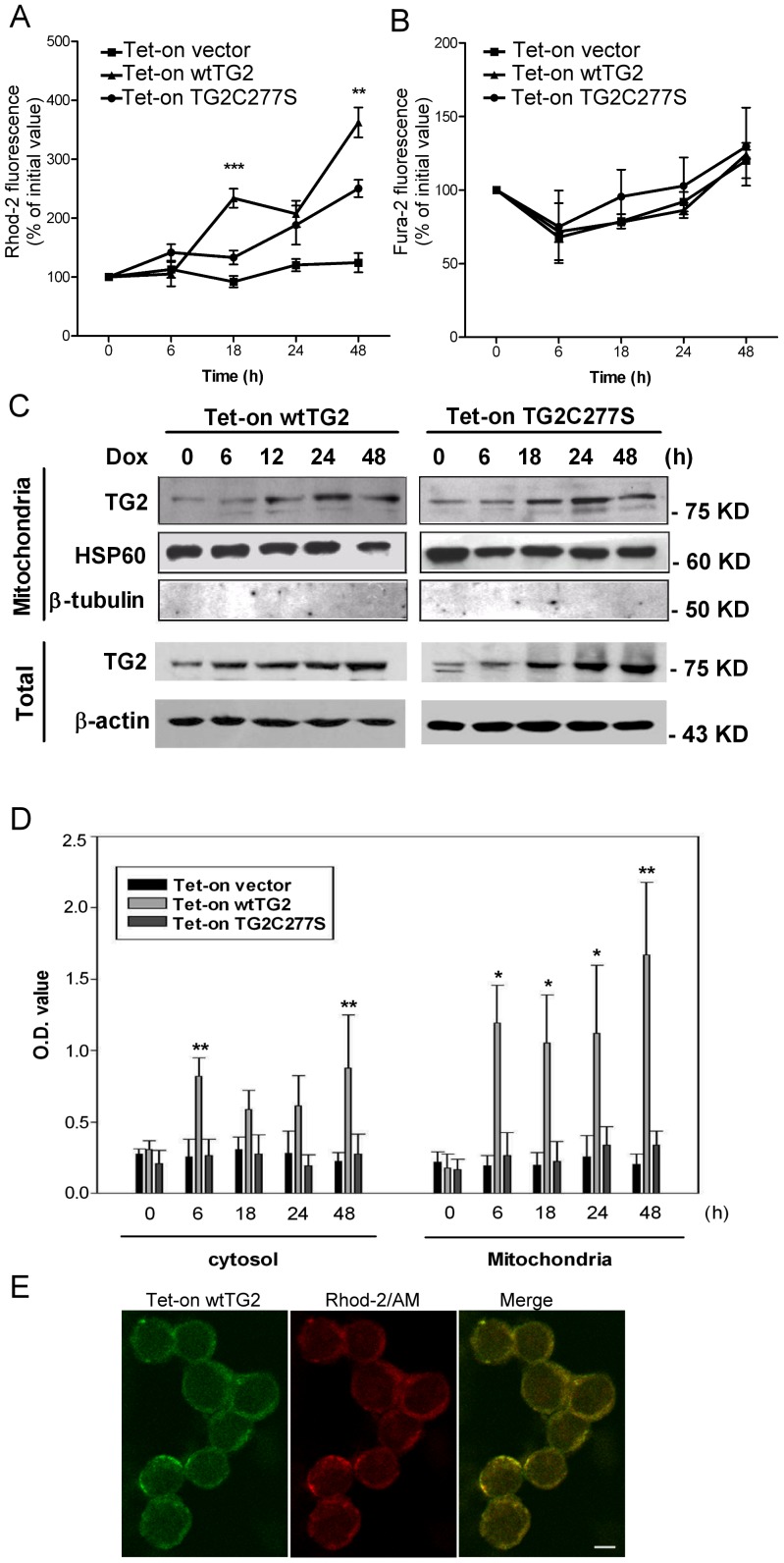
Overexpressed wtTG2 appears in the mitochondria and induces enhanced mitochondrial Ca^2+^ accumulation. (A) Time dependent changes in the mitochondrial Ca^2+^ concentrations of Tet-on vector, Tet-on wtTG2 and Tet-on TG2C277S cells following Dox (50 µM) treatment detected by Rhod-2/AM. (B) Time dependent changes in the cytosolic Ca^2+^ concentrations of Tet-on vector, Tet-on wtTG2 and Tet-on TG2C277S cells following Dox (50 µM) treatment detected by Fura-2/AM. (C) Time dependent changes in the mitochondrial and cytoplasmic TG2 expressions of Tet-on wtTG2 and Tet-on TG2C277S cells following Dox (50 µM) treatment detected by Western blot analysis. Mitochondrial HSP60 and cytoplasmic β-actin were used as loading controls; while β-tubulin was used to check for cytoplasmic contamination of mitochondria. (D) Time dependent changes in the mitochondrial and cytoplasmic TG2 activities of Tet-on vector, Tet-on wtTG2 and Tet-on TG2C277S cells following Dox (50 µM) treatment. (E) Confocal images taken at 16 h following Dox treatment show Tet-on wtTG2 cells expressing TG2 (green) colocalized with the mitochondrial calcium indicator Rhod-2/AM (red). Scale bar = 5 µM. All the data presented represent mean±S.D. of at least three determinations. Significantly different from the TG2C277S cell line detected at the same time point (*P<0.5; **P<0.01; ***P<0.001).

Ca^2+^ is known to accumulate preferentially in the mitochondria in Jurkat T cells [22]. Enhanced apoptosis of wtTG2 expressing Jurkat T cells was indeed accompanied by an enhanced intra-mitochondrial Ca^2+^ concentration detected by following changes in the intra-mitochondrial Ca^2+^ concentration as a function of time with the help of the Ca^2+^-sensitive fluorescent indicator Rhod-2 (Figure 2A). Following Dox addition mitochondrial Ca^2+^ concentration increased in TG2X expressing cells as well, but a more significant increase was found in wtTG2 expressing cells. The enhanced mitochondrial Ca^2+^ concentration does not seem to be the result of an enhanced cytosolic Ca^2+^ concentration and a consequently enhanced Ca^2+^ uptake, since the cytosolic Ca^2+^ concentration detected by Fura-2-AM did not change dramatically during the same time in any of the cell lines following Dox exposure (Figure 2B). These data indicate that crosslinking activity of TG2 might promote mitochondrial calcium uptake during apoptosis.

Previous studies have shown that TG2 can be localized in various organelles in the cell including mitochondria [3]. To test whether TG2 is also expressed in the mitochondria of T cells subcellular fractionation was performed. Following Dox exposure expression of both the basal and the induced TG2 could be detected in the mitochondrial fractions of Tet-On wtTG2 and Tet-On TG2C277S cells by Western blot analysis (Figure 2C). Transglutaminase enzyme activity increased also rapidly in the mitochondrial fraction of wtTG2 cells, while it was not significantly induced in the control or in the TG2C277S cells (Figure 2D). In addition, detected by confocal microscopy, in wtTG2 overexpessing cells TG2 co-localized with the mitochondrial Ca^2+^ indicator Rhod-2/AM (Figure 2E).

### Transglutaminase 2 enhances mitochondrial Ca^2+^ uptake indirectly by promoting Ca^2+^ release from the ER

**Figure 3:**
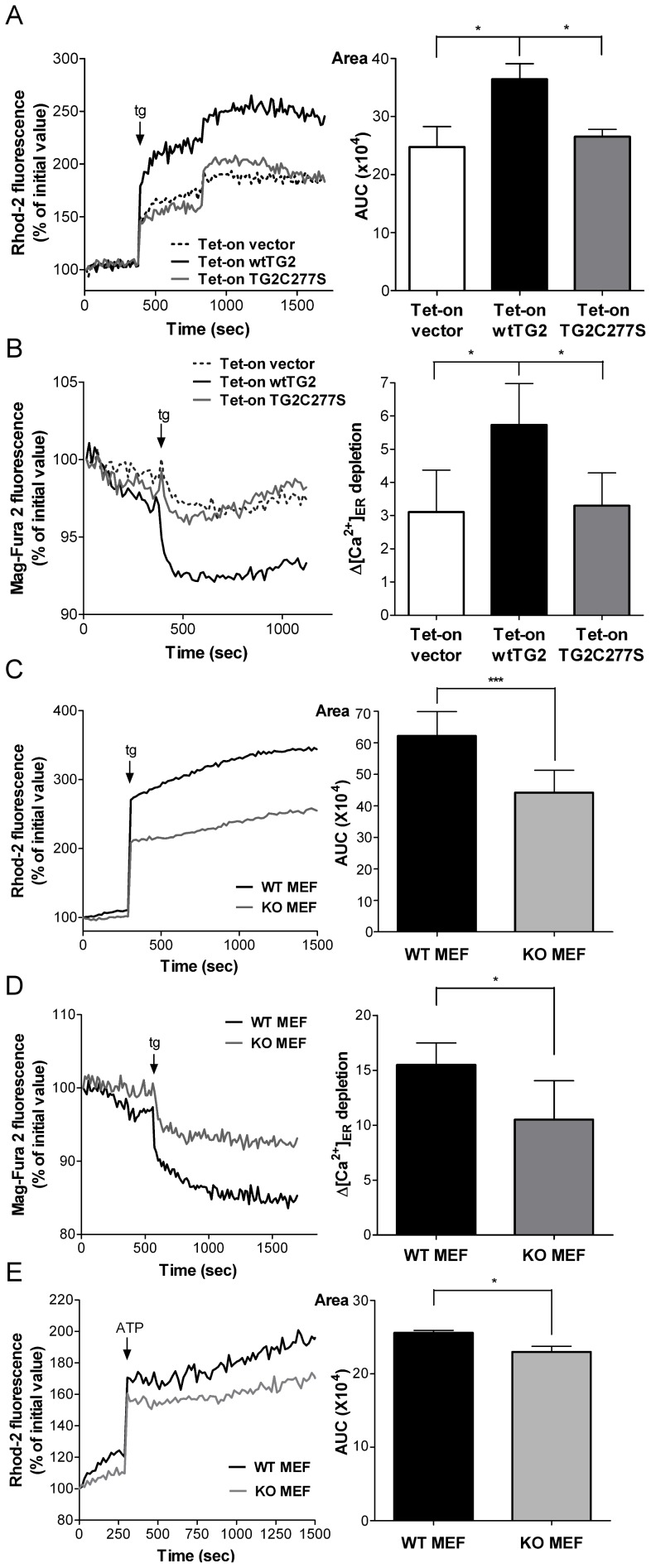
Transglutaminase 2 enhances both the Ca^2+^ release from the endoplasmic reticulum and the mitochondrial Ca^2+^ uptake. Tet-on vector, Tet-on wtTG2 and Tet-on TG2C277S cells treated with Dox (50 µM) for 18 h were exposed to 5 µM thapsigargin (tg), and changes in the Ca^2+^ concentrations in the mitochondria (A) or in the ER (B) were monitored as described in the Materials and Methods. Wild type and TG2KO MEF cells were exposed to 5 µM thapsigarginin Ca^2+^ free medium and changes in the Ca^2+^ concentrations in the mitochondria (C) or in the ER (D) were monitored as described in the Materials and Methods. (**E**) Wild type and TG2 KO MEF cells were exposed to 500 µM ATP in Ca^2+^-free medium, and changes in the intra-mitochondrial Ca^2+^ concentrations were monitored as described in the Materials and Methods. *Left panels*, Representative kinetic average changes in mitochondrial or ER Ca^2+^signals induced by thapsigarginor ATP over time are shown. *Right panels*, Areas, statistical evaluation of integrated Ca^2+^ responses are shown. AUC, area under the curve. These data are representative of at least three experiments and shown as mean ± SD. *P<0.05; ***, *P*<0.001.

The cross-linking activity of TG2 is activated by elevations in intracellular Ca^2+^ concentrations. To confirm further that the transamidation activity of TG2 indeed influences intra-mitochondrial Ca^2+^ homeostasis, all the three types of transfected Jurkat cells treated with Dox for 18 hours were exposed to thapsigargin, an irreversible SERCA pump inhibitor [23], with the aim of promoting Ca^2+^ release from the ER and subsequently activate TG2 cross-linking activity. Following thapsigargin exposure, intra-mitochondrial Ca^2+^ was immediately elevated in all the three cell lines, but it reached significantly higher levels in the wtTG2 expressing cells than in the others (Figure 3A).

The enhanced mitochondrial Ca^2+^ concentration achieved by the overexpression of TG2 can be the result either of an enhanced mitochondrial Ca^2+^ uptake, or of an enhanced Ca^2+^ release from the ER. To test this latter alternative, Ca^2+^ release from the ER was also determined following thapsigargin exposure by detecting changes in the high Ca^2+^ concentration of the ER by using the low affinity Ca^2+^ fluorescent dye Mag-Fura 2-AM (Kd≈50 µM) [24]. As shown in Figure 3B, wtTG2 cells released Ca^2+^ faster from the ER in the presence of thapsigargin, than TG2C277S or vector cells did, indicating that TG2 might act primarily on the ER.

Interestingly, a similar difference in the thapsigargin-induced Ca^2+^ release from the ER and in the increase of the intra-mitochondrial Ca^2+^ concentration was found, when mouse embryonic fibroblasts (MEFs) isolated from wild type and TG2 knock out mice were exposed to thapsigargin (Figure 3C and D). A similar difference in the mitochondrial Ca^2+^ uptake was found also, when these cells were stimulated with adenosine 5′-triphosphate (ATP), the P2Y receptor agonist that causes release of Ca^2+^ from the ER via Ins_3_P receptors [25] (Figure 3E).These data indicate that TG2 might affect intra-mitochondrial Ca^2+^ homeostasis not only when it is overexpressed, but also at physiological levels, and not only when thapsigargin is added, but also after applying a physiological stimulus.

**Figure 4:**
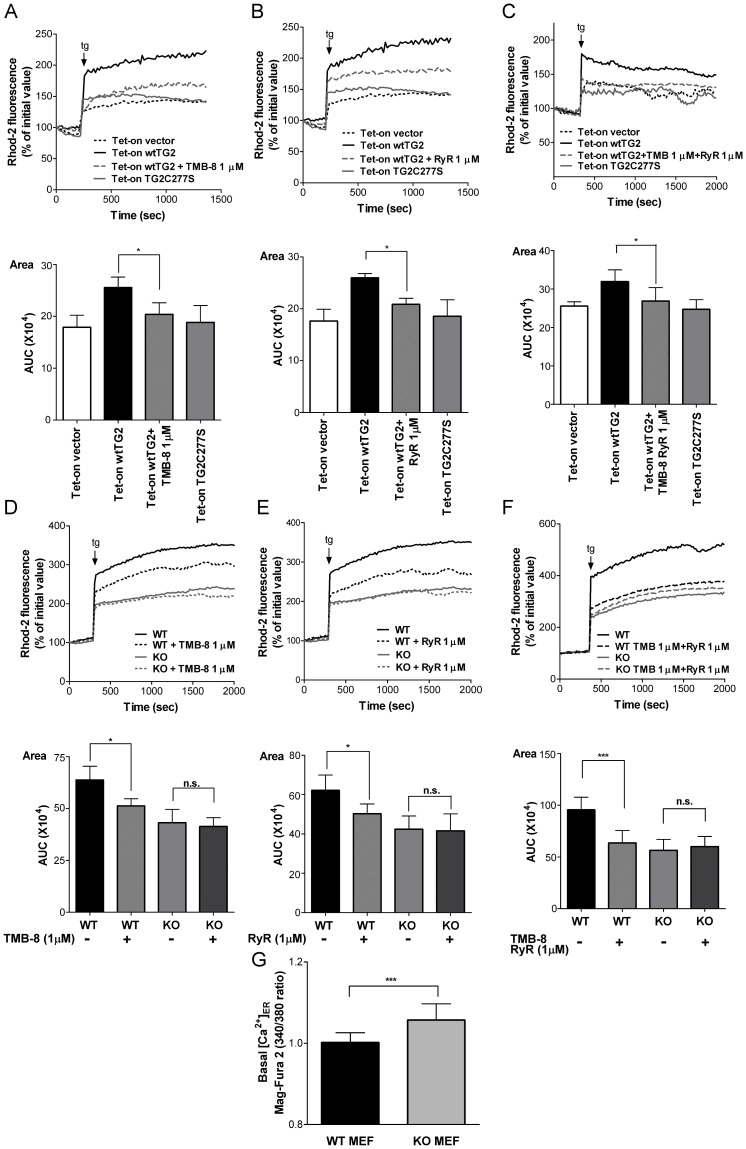
Transglutaminase 2 affects both Ins_3_P and ryanodine sensitive receptors to enhance mitochondrial Ca^2+^ uptake. (A–C) Representative recordings of thapsigargin-induced intra-mitochondrial Ca^2+^ elevations in Tet-on cells treated previously with Dox (50 µM) for 18 h, in the presence or absence of 1 µM TMB-8, an Ins_3_P receptor antagonist, 1 µM ryanodine, or both. Tg response of Tet-on vector and Tet-on TG2C277S cells treated with Dox (50 µM) for 18 h is also shown. (D–F) Representative recordings of tg-induced intra-mitochondrial Ca^2+^ elevations in in WT MEF and TG2 KO MEF cells in the presence or absence of 1 µM TMB-8, 1 µM Ryanodine or both. *Top panels*, Representative kinetic average changes in mitochondrial Ca^2+^signals induced by tg (5 µM) over time are shown. *Bottom panels*, Areas, statistical evaluation of integrated Ca^2+^ responses are shown. AUC, area under the curve. (G) Resting value of Mag-Fura 2 ratios (340/380) were measured in Mag-fura-2/AM loaded WT MEF and TG2 KO MEF cells. All the presented data are representative of at least three experiments and are shown as mean ± SD. *, *P*<0.05; ***, *P*<0.00; n.s., no significance.

Under resting conditions the net release of Ca^2+^ from the ER and the consequent mitochondrial Ca^2+^ uptake and intra-mitochondrial Ca^2+^ concentration result from a balance between the activity of the SERCA pumps, which import Ca^2+^ into the ER, and the total release of Ca^2+^ from the ER. Addition of thapsigargin artificially shifts this balance towards to the total ER Ca^2+^ release which results in a consequently enhanced mitochondrial Ca^2+^ uptake. ER expresses Ins_3_P and ryanodine sensitive receptors to release Ca^2+^ for signaling purposes [24]. To identify which of these Ca^2+^ channels might be affected by the transamidating activity of TG2, thapsigargin-induced mitochondrial Ca^2+^ concentration changes were detected in the presence of TMB-8, an antagonist of the Ins_3_P receptor [26], and/or ryanodine, respectively. Pre-treatment of wtTG2 cells with either TMB-8 (Figure 4A) or ryanodine (Figure 4B) for 30 min resulted in suppression of thapsigargin-induced elevation in the intra-mitochondrial Ca^2+^ concentration. When both inhibitors were applied together, the difference in the thapsigargin-induced mitochondrial Ca^2+^ uptake between wtTG2 and TG2X expressing cells completely disappeared (Figure 4C). These observations were confirmed by using wild type and TG2 knock out MEFs. While both TMB-8 and ryanodine were capable of inhibiting the thapsigargin-induced increase in the intra-mitochondrial Ca^2+^ concentration in wild type MEFs (Figure 4D and E), none of them had an effect when the thapsigargin response was investigated in the knock out cells (Figure 4D and E). Application of both inhibitors to wtTG2 cells completely abolished the observed difference in the thapsigargin response of wild type and TG2 knock out MEFs (Figure 4F). Theoretically TG2 could act via enhancing Ca^2+^ levels in the ER and thus promoting Ca^2+^ release via all Ca^2+^ channels of the ER in a Ca^2+^ concentration dependent manner. However, no decrease in the ER Ca^2+^ concentration was found in the knock out fibroblasts in the absence of TG2 (Figure 4G) indicating that TG2 acts primarily on Ins_3_P and ryanodine sensitive receptors to promote Ca^2+^ release from the ER.

### Overexpressed TG2 cross-links RAP1GDS1

To determine which protein mediates the effect of TG2 on the Ca^2+^ homeostasis, JK-Tet-On wtTG2 and TG2C277S cells were exposed to Dox for 18 hrs and their protein expression levels were compared after two-dimensional gel electrophoresis. Several spots, the level of which were either increased or decreased in the wtTG2 expressing cells as compared to the TG2C277S cells, were selected and their identity was determined by LC/MS-MS (Table I). From the 19 identified proteins we selected RAP1GDS1, an unusual guanine exchange factor acting on numerous small GTPases [19], as the candidate for further studies, because two small GTPases (RAP1 and RAP2) reported to be regulated by it, have already been linked to the regulation of ER Ca^2+^ homeostasis [27]–[29].

**Table 1:** List of proteins identified in Tet-on wtTG2 cells by LC/MS-MS.

Sample no.	regulation	Protein name	Molecular size	Accession number
1	down	enolase 1	47 kDa	4503571
2	up	tubulin, beta, 5	49 kDa	21361322
3	down	glyceraldehyde-3-phosphate dehydrogenase	36 kDa	7669492
4	down	Vimentin	53 kDa	4507895
6	up	heat shock 70 kDa protein 2	69 kDa	13676857
7	up	alpha 1 actin precursor; alpha skeletal muscle actin	42 kDa	4501881
8	down	Triosephosphate isomerase (TIM)	27 kDa	41058276
9	down	similar to Triosephosphate isomerase (TIM)	27 kDa	41058276
10	down	similar to Triosephosphate isomerase (TIM)	27 kDa	41058276
11	down	keratin 10	59 kDa	40314192
12	down	glyceraldehyde-3-phosphate dehydrogenase	36 kDa	7669492
13	down	glyceraldehyde-3-phosphate dehydrogenase	36 kDa	7669492
14	down	glyceraldehyde-3-phosphate dehydrogenase	36 kDa	7669492
15	up	Cryptic	25 kDa	14211837
16	down	enolase 1	47 kDa	4503571
17	down	enolase 1	47 kDa	4503571
18	down	Williams Beuren syndrome chromosome region 24	49 kDa	51465954
19	down	RAP1,GTP-GDP dissociation stimulator 1 (RAP1GDS1)	60 kDa	20070314

**Figure 5:**
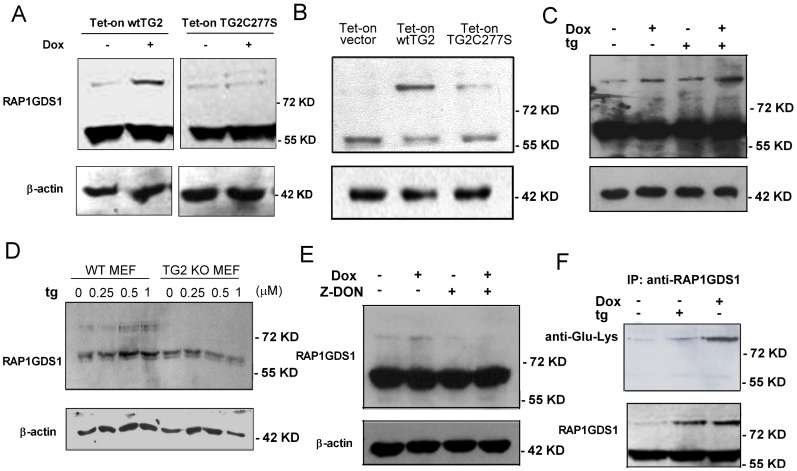
Ca^2+^-activated TG2 cross-links RAP1GDS1. Expression of RAP1GDS1 analyzed by Western blotting in Tet-on wtTG2 and Tet-on TG2C277S cells treated or not with Dox (50 µM) for (A) 6 or (B) 18 hours. (C) Increasing amounts of cross-linked RAP1GDS1 in Tet-on wtTG2 cells treated with tg (5 µM) for 15 min, Dox for 6 hours and subsequently exposed to tg (5 µM) for 15 min analyzed by Western blotting. (D) Increasing amounts of cross-linked RAP1GDS1 in WT but not in TG2 KO MEF cells exposed to increasing concentrations of tg for 15 min analyzed by Western blotting. In all these experiments β-actin was used as loading control. (E) Tet-on wtTG2 cells were treated either with tg (5 µM) for 15 min, or with Dox for 6 hours. RAP1GDS1 was immunoprecipitated from the cell lysate and probed with anti-ε(γ-glutamyl) lysine isopeptide antibodies.

To prove that RAP1GDS1 is indeed a TG2 substrate, the time-dependent expression of the protein was followed by Western blot analysis in both the wtTG2 and in the TG2C277S cells following Dox exposure. As shown in Figure 5A, RAP1GDS1 appeared in two bands in Jurkat cells, with one band appearing at 57 kD and another one close to 100 kD molecular weight. In non-treated cells the upper band was very faint, but the intensity of it significantly increased by 6 hr in the wtTG2 overexpressing cells, when TG2 was already induced in these cells following Dox exposure. No similar increase in the intensity of the upper band was detected at this early time point in the cross-linking mutant TG2 expressing cells (Figure 5A). Tested however at 18 hr following Dox treatment, the upper band became stronger in the TG2X expressing cells as well (Figure 5B) indicating that the endogenously expressed TG2 can also initiate the formation of high molecular weightRAP1GDS1, when apoptosis is detectable in the cell line. In addition, formation of the higher amount of high molecular weight RAP1GDS1 was accompanied with the disappearance of the low molecular weight RAP1GDS1 indicating a possible conversion of low molecular weight RAP1GDS1 to the higher molecular weight one. Addition of thapsigargin was capable of further inducing the intensity of the upper band within 15 min tested in the wtTG2 expressing cells exposed to Dox for 6 h indicating that the upper band formation is fast and Ca^2+^-dependent (Figure 5C).

Since it was recently reported that C277S is not only impaired in its transamidase activity, but GTP-binding is also significantly impaired [30], we decided to prove that the upper band of RAP1GDS1 appears indeed as a result of the cross-linking activity of TG2. For this reason wtTG2 expressing Jurkat cells were preincubated with Z-DON, an inhibitor of TG2 transamidase activity, which can act intracellularly. As seen in Figure 5D, addition of Z-DON prevented formation of the high molecular band RAP1GDS1 in Dox exposed cells.

To prove further that the upper band of RAP1GDS1 appears in a TG2-dependent manner and can be detected in other cell types as well, RAP1GDS1 protein levels were also determined in wild type and TG2 knock out MEFs following 15 min of thapsigargin exposure. As seen in Figure 5E, similar to Jurkat T cells, wild type MEFs already expressed the high molecular weight RAP1GDS1. But cells lacking TG2 did not, indicating that physiological levels of TG2 maintain a certain level of high molecular weight RAP1GDS1. Addition of thapsigargin induced further the amount of high molecular weight RAP1GDS1 in a dose dependent manner in wt MEFs, while no high molecular weight bands were detectable in the knock out cells.

To prove that the high molecular band RAP1GDS1 is actually a cross-linked product of TG2, wtTG2 expressing Jurkat cells were exposed to thapsigargin either alone or following 6 h Dox treatment. RAP1GDS1 was than immunoprecipitated with anti-RAP1GDS1 antibody from the cell lysate and was stained for anti-ε(γ-glutamyl) lysine isopeptide. As seen in Figure 5F, both the low and the high bands of RAP1GDS1 could be immunoprecipitated from the cell lysates, and the high bands were detected also by the anti-ε(γ-glutamyl) lysine isopeptide antibodies. These data indicate that Rap1GDS1 is a substrate for the transamidating activity of TG2, that endogenous TG2 maintains a basal level of cross-linked RAP1GDS1, and that the increase in either the TG2 or the intracellular Ca^2+^ levels can enhance the TG2-mediated cross-linking of RAP1GDS1.

### RAP1GDS1 mediates the effect of TG2 on Ca^2+^ homeostasis

**Figure 6:**
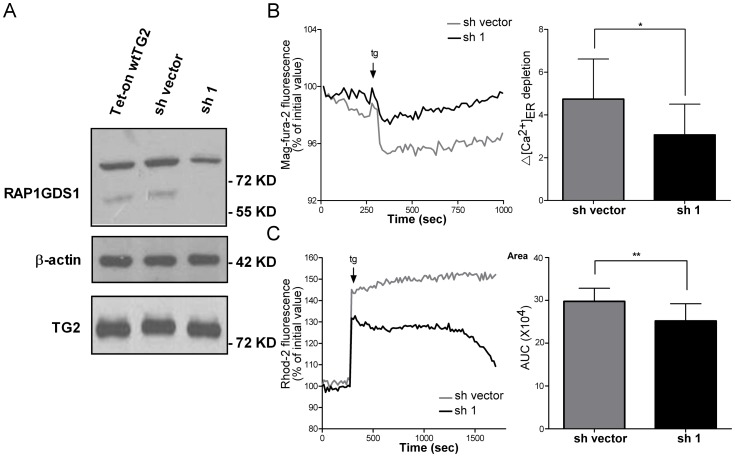
RAP1GDS1 mediates the enhancing effect of TG2 on the calcium release from ER and on the subsequent mitochondrial Ca^2+^ uptake. (A) RAP1GDS1 and TG2 protein expression levels detected by Western blotting in Tet-on wtTG2, Tet-on wtTG2 with sh vector and Tet-on wtTG2 with shRAP1GDS1 (sh1) cells following 18 hours Dox (50 µM) treatment. β-actin was used as loading control. Tet-On wtTG2 with sh vector and sh1 cells treated with Dox for 18 hours were exposed to 5 µM thapsigargin. (B) A representative recording of the tg-induced Ca^2+^ release from the ER recorded by Mag-Fura-2/AM fluorescence is shown. *Right panel*, statistical evaluation of the tg-induced ER Ca^2+^ depletion. (C) A representative recording of the tg-induced intra-mitochondrial Ca^2+^ changes is shown. *Right panel*, Area, statistical evaluation of integrated Ca^2+^ response. AUC, area under the curve. These data are representative of at least three experiments and shown as mean ± SD. *, *P*<0.05; **, *P*<0.01.

To prove that RAP1GDS1 might mediate the effect of TG2 on Ca^2+^ homeostasis, RAP1GDS1 was silenced by lentiviral shRNA in the wtTG2 expressing Jurkat T cells. Figure 6A demonstrates the amount of cross-linked RAP1GDS1 protein in the various Jurkat T cell lines after 18 hrs Dox treatment. The lentiviral infection with the control RNA alone (sh vector cells) slightly increased the amount of cross-linked RAP1GDS1 following Dox treatment as compared to the lentiviral free wtTG2 cells, but this was not related to an increase in the TG2 levels. In the presence of the targeted shRNA, however, the lower band completely disappeared and the amount of high molecular weight RAP1GDS1 was also significantly decreased. Testing these 2 latter cell lines we found that the decrease in the RAP1GDS1 levels significantly attenuated the TG2-mediated Ca^2+^ release from the ER (Figure 6B) and the consequent mitochondrial Ca^2+^ uptake (Figure 6C) following thapsigargin exposure. All together these data indicate that RAP1GDS1 indeed might mediate the effect of TG2 on Ca^2+^ homeostasis.

### Cross-linked RAP1GDS1 is found in the ER

**Figure 7:**
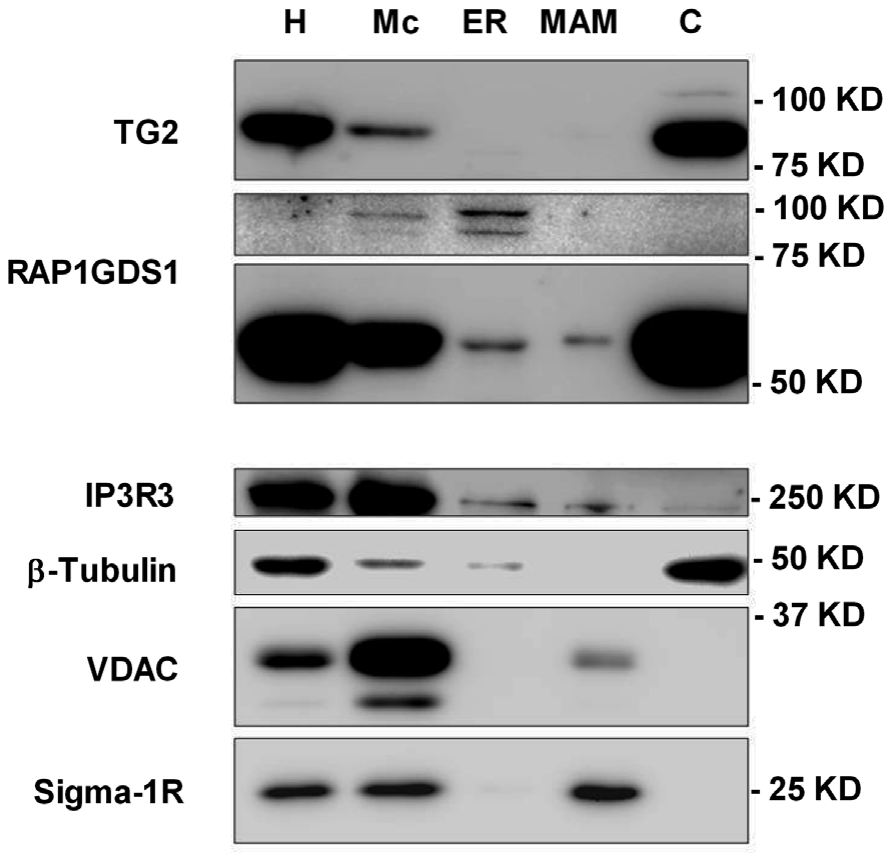
Intracellular distributions of TG2 and RAP1GDS1 in mitochondria, ER, MAM and cytosol. H: homogenate; Mc: crude mitochondria; ER; MAM: mitochondria-associated membrane; C: cytosol. Ten µg of protein components of subcellular fractions prepared from Jurkat Tet-On cells were loaded on 10% SDS-PAGE and transferred to PVDF membrane for standard western blotting. The presence of TG2 and RAP1GDS1 were shown using specific monoclonal antibodies. Marker proteins indicate mitochondria (VDAC), ER (IP3R3), MAM (Sigma-1R) and cytosol (β-tubulin).

To decide in which cellular compartment TG2 can cross-link RAP1GDS1, Jurkat cells were fractionated and tested for the location of TG2, RAP1GDS1 and the cross-linked RAP1GDS1. As shown in Figure 7, under basal conditions TG2 was localized in the cytosol and in the mitochondria, but clearly no TG2 expression was seen in the ER or its MAMs. The monomeric form of RAP1GDS1 was detected in each fraction, while the cross-linked RAP1GDS1 was clearly associated with the ER, but not with its MAMs. Some cross-linked RAP1GDS1 could be detected in the crude mitochondrial fraction as well. However, the crude mitochondrial fraction contained a large amount of Ins_3_P receptor indicating that it was contaminated with the ER. Since the ratio of monomeric versus cross-linked RAP1GDS1 was much higher in the ER than in the mitochondria, the cross-linked RAP1GDS1 might have come from the ER contamination, while the monomeric RAP1GDS1 might also be expressed by the mitochondria.

## Discussion

Recent studies using electron tomography techniques revealed the presence of overlapping regions between ER and mitochondria separated by a minimum distance of 10–25 nm that allows the direct physical association of ER proteins with components of the outer mitochondrial membrane [31]. These zones were identified as MAMs and play a pivotal role in the highly efficient transmission of Ca^2+^ from the ER to the adjacent mitochondrial network that under basal conditions stimulates oxidative metabolism, while during apoptosis sensitizes mitochondria for the death-inducing stimuli [32]. Ca^2+^ release is therefore considered an intrinsic mechanism that favors apoptosis [33].

In the present study we found that if overexpressed both wtTG2 and its cross-linking mutant induce apoptosis in Jurkat T cells, but the wtTG2 enzyme is more effective in the cell death induction. Previous studies have already indicated that overexpressed TG2 is capable of inducing cell death in certain cell types [34]. Though several mechanisms related to the crosslinking activity of TG2 through which the enzyme can contribute to cell death induction have been identified [12], [13], some other biological activities of TG2, such as its protein kinase activity [17] or its BH3 only domain [3] alone might be sufficient to induce cell death, when the protein is overexpressed. A new difference we found between the wt and TG2X expressing cells was a significantly higher intra-mitochondrial Ca^2+^ concentration in wtTG2 expressing cells. Following induction of wtTG2 not only the basal intra-mitochondrial Ca^2+^ concentration increased, but these cells responded with an enhanced mitochondrial Ca^2+^ uptake to the administration of thapsigargin, a SERCA pump inhibitor. The response seemed to be the consequence of an enhanced Ca^2+^ release from the ER in the presence of higher levels of wtTG2. Similar was the finding, when the thapsigargin and the ATP response of wild type and TG2 null MEFs were compared indicating that physiological levels of TG2 and physiological stimuli regulating the Ca^2+^-dependent crosslinking activity of TG2 are also able to affect intra-mitochondrial Ca^2+^ homeostasis. Using various Ca^2+^ channel inhibitors we found that TG2 acts on both Ins_3_P and ryanodine sensitive receptors to promote Ca^2+^ release from the ER. Since in TG2 null cells the thapsigargin-response was not effected either by the Ins_3_P receptor inhibitor or by ryanodine, but their intra-mitochondrial Ca^2+^ also increased upon thapsigargin exposure, our data imply that there must be a third type of Ca^2+^ channel on the ER of Jurkat T cells that releases Ca^2+^ and the activity of which is not affected by TG2. It has been suggested previously that this channel might be the translocon complex, through which ER leaks Ca^2+^ continuously [35]. Based on our data we propose that following thapsigargin addition ER releases Ca^2+^ first, possibly via the translocon complex, and the released Ca^2+^ activates the cross-linking activity of TG2. Activated TG2 than by using its cross-linking activity induces a signaling pathway that either sensitizes the Ins_3_P and ryanodine sensitive receptors to their endogenous ligands being present at low concentrations, or triggers enzymes (e.g. phospholipase C, ADP-ribosyl cyclase), which form seconder messengers for these receptors. Since ATP in fibroblasts can also trigger the same response, our data indicate that any signal that raises intracellular Ca^2+^ and triggers TG2 transamidase activity will amplify the Ca^2+^ signal by promoting ER Ca^2+^ release. As apoptosis is usually accompanied by increases in the intracellular Ca^2+^ concentrations, we propose that the crosslinking activity of TG2 might contribute to the apoptosis initiation by amplifying these Ca^2+^ signals.

We identified RAP1GDS, an unusual guanine exchange factor acting on various small GTPases [19], as a possible mediator of the TG2-induced events. TG2 was already shown to act on small GTPases [36], [37], but this is the first evidence that it might act also on a guanine exchange factor to regulate signaling events. It is interesting to speculate that the cross-linking might stabilize that conformation of RAP1GDS1, which is able to interact and activate its downstream target small GTPase. We have so far not identified the downstream small GTPase of RAP1GDS1, but two of its known target proteins, RAP1 and RAP2 were already connected to the ER Ca^2+^ homeostasis [27]–[29].

TG2 and RAP1GDS1 are found together in both the cytosol and in the mitochondria, but under basal conditions cross-linked RAP1GDS1 is located only in the ER. Since basal cross-linked RAP1GDS1 levels can be detected in TG2 expressing wild type Jurkat and MEF cells, but not in the knock out cells, and MEFs respond to ATP in a TG2-dependent manner, though it is debated [38], our data provide an additional proof for the possibility of TG2 to be intracellularly activated, where and when the intracellular Ca^2+^ concentrations rise. Because of the tight location of the ER and mitochondria, it is interesting to speculate that mitochondrial TG2, sensing Ca^2+^ fluxes from the ER, might cross-link RAP1GDS1 located in the ER membranes which, in turn, triggers a signaling pathway that forms an amplifying loop for the ER-derived Ca^2+^ signals.

## Conclusions

**Figure 8:**
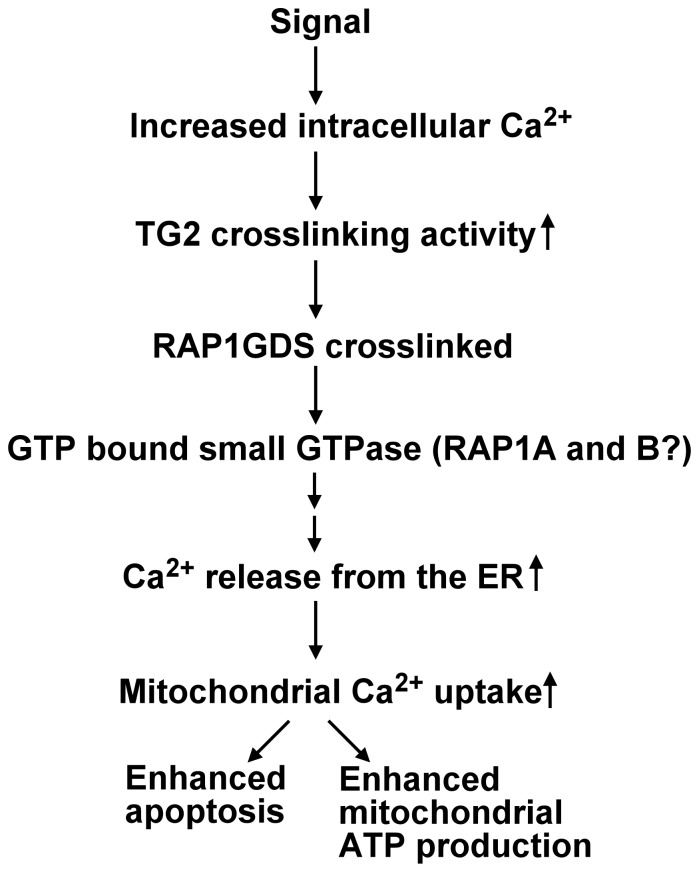
Proposed mechanism through which the crosslinking activity of TG2 might amplify Ca^2+^ signals in the cell. Increases in intracellular Ca^2+^ concentrations trigger the crosslinking activity of TG2. TG2 crosslinks RAP1GDS1, which in turn facilitates the exchange of GDP to GTP on its target GTPase protein. This small GTPase initiates a not yet characterized signalling pathway, which enhances Ca^2+^ release from the ER and the consequent Ca^2+^ uptake of the mitochondria. High amounts of mitochondrial Ca^2+^ sensitize for apoptosis, while physiological amounts enhance mitochondrial ATP production.

In the present paper we have demonstrated the TG2, if overexpressed, can induce apoptosis in T cells and both its crosslinking and crosslinking-unrelated activities contribute to the phenomenon. We identified a new signaling pathway related to the calcium regulated crosslinking activity of TG2 that involves the unusual guanine exchange factor RAP1GDS1, and promotes the Ca^2+^ release from the ER via both the Ins_3_P receptor and ryanodine sensitive channels leading to an enhanced mitochondrial Ca^2+^ uptake (Figure 8). Since enhanced mitochondrial Ca^2+^ concentrations were shown to sensitize mitochondria for the action of pro-apoptotic factors [8], [9], this signaling pathway might contribute to the initiation of the apoptotic process. Since under basal conditions mitochondrial Ca^2+^ uptake regulates the rate of mitochondrial ATP synthesis [33], we propose that the loss of this amplifying loop might contribute to the impairment of ATP synthesis in the heart of TG2 null mice [39], or to the development of MODY type of diabetes mellitus [40], which is believed to be the result of an impaired ATP synthesis in the pancreatic β-cells [41]. Studies are in process to investigate these possibilities.

## Methods

### Cell culture

The Jurkat (JK), JK-Tet-On Vector, JK-Tet-On wtTG2 and JK-Tet-On TG2C277S cells were grown in 90% RPMI 1640 and 10% fetal bovine serum (FBS) obtained from Gibco BRL (Grand Island, NY) at a temperature of 37°C under a humidified and 5% CO_2_ atmosphere. Wild type (WT) and TG2 knock out (KO) mouse embryonal fibroblasts (MEF) derived from wild-type and TG2 knock out mice were kindly provided by Professor Mauro Piacentini, University Tor Vergata [42]. MEFs were cultured in 90% DMEM and 10% fetal FBS at a temperature of 37°C under a humidified and 5% CO_2_ atmosphere.

### Human wtTG2 and TG2C277S gene sub-cloning and cell transfection

The entire coding sequence of human wild type (wtTG2) and mutant TG2 (TG2C277S), a variant containing a point mutation in the active site (TGC→AGC, Cys277→Ser), cDNA were kindly provided by Professor Peter Davies, Houston, TX. Briefly, wtTG2 and TG2C277S cDNA was amplified using a sense primer consisting of a 5′*Mlu*I site (underlined) and a 21-nt sequence: ACGCGTATGGCCGAGGAGCTGGTCTTA, an anti-sense primer consisting of a 5′*Sal*I site (underlined) and a 18-nt sequence: GTCGACTTAGGCGGGGCCAATGAT. The polymerase chain reaction (PCR) product was ligated into the yT&A vector and transformed into *E. coli* strain DH5α. The bacteria was grown for 24 hours at 37°C, eluted plasmids and the amplified plasmid was digested with *Mlu*I-*Sal*I and sub-cloned into the *Mlu*I-*Sal*I site of pTRE2hgy for the Tet-On system (BD Biosciences Clontech, San Jose, CA), and named Tet-On wtTG2 and Tet-On TG2C277S. TRE2hyg-wtTG2, pTRE2hyg-TG2C277S and pTRE2hyg-vector only were transfected into JK-Tet-On system cells (BD Biosciences, Clontech) using electroporation with a MP-100 Microporator (Degital Bio Technology) with the parameters 1,300 V, two pulses, and 20 ms according to the manufacturer's instructions. Stably transfected cells were selected with the antibiotic hygromycin B (400 µg/ml). After approximately 3 weeks, hygromycin-resistant clones were screened for protein expression and enzymatic activity by Western blot and colorimetric assay of transglutaminase activity, respectively. *In vitro* promoter induction of Tet-On cell system was accomplished with the addition of 50 µM doxycycline to the growth medium.

### Enzyme activity assay for transglutaminase 2

Transglutaminase enzyme activity was assayed at 37°C as described previously [43]. Microplates were coated with 20 mg/ml of *N*, *N*′-dimethylcasein and blocked with nonfat dry milk (0.5% in 0.1 M Tris-HCl, pH 8.5). Twenty micrograms of cell lysates were extracted and sonicated for 10 seconds in 5 mM Tris-HCl, pH 7.4, 0.25 M sucrose, 0.2 mM MgSO_4_, 2 mM dithiothreitol (DTT), 0.4 mM phenylmethylsulfonyl fluoride (PMSF), and 0.4% Triton X-100, and were then loaded onto the previously coated microplate. The lysates were incubated for 1 hour in 100 mM Tris, pH 8.5, 5 mM CaCl_2_, 10 mM DTT, and 0.5 mM 5-(biotinamido) pentylamine (BP) (Pierce Biotechnology Inc., Rockford, IL), and detection of the bounded BP was performed at OD of 405.

### Cell viability

Cell viability was assessed by the mitochondrial-dependent reduction of 3-(4, 5-dimethylthiazol-2-yl)-2, 5-diphenyl tetrazolium bromide (MTT) to purple formazan. For MTT assay, Jurkat T cells (5×10^4^) were added in 96-well plates. After incubation, 50 µl MTT solutions (1.1 mg/ml) were added to each well and incubated for an additional 4 hours. After centrifugation, the supernatant was removed from each well and was replaced by 150 µl DMSO to dissolve the colored formazan crystal produced from MTT. OD values of the solutions were measured at 540 nm by a plate reader. Cell viability was expressed as percentage of viable cells at a given time as compared to the number of viable cells (100%) at the time point of doxycycline addition.

### Detection of apoptotic cells by Annexin V-FITC/propidium iodide staining

The Jurkat cell lines were treated with 50 µM Dox for the indicated time points and were than stained with the Annexin V-FITC Apoptotis Detection Kit (Sigma-Aldrich, Budapest) according to the manufacturer's instructions. In each study 10,000 events (cells) were counted. Data were analyzed by using WinMDI software.

### Detection of apoptotic cells by flow cytometry

For detecting apoptotic cells in sub-G1, 1×10^6^ cells were harvested, washed with 0.2 ml of PBS and fixed in 0.8 ml of ice-cold 99% ethanol at −20°C overnight. The cell pellets were collected by centrifugation, and resuspended in 1 ml of hypotonic buffer (0.5% Triton X-100 in PBS containing 0.5 µg/ml RNase A), and incubated at 37°C for 30 minutes. Subsequently, 1 ml of propidium iodide solution (10 µg/ml) was added and the mixture was allowed to stand on ice for 30 minutes. The stained cells were analyzed for cell cycle distribution in a FACSCAN laser flow cytometry (Becton Dickenson, San Jose, CA).

### DNA fragmentation assay

Cells were harvested and lysed overnight in a digestion buffer (0.5% sarkosyl, 0.5 mg/ml proteinase K, 50 mM Tris–HCl, pH 8.0 and 10 mM EDTA) at 55°C. Subsequently, cells were treated with 0.5 µg/ml RNase A for 2 hours. The genomic DNA was extracted by phenol/chloroform/isoamyl alcohol extraction and analyzed by gel electrophoresis using 2% agarose.

### Western blotting for the detection of TG2 or RAP1GDS1

To extract separated mitochondrial and cytosolic proteins, cells were washed once with PBS, and isolated using the Pierce mitochondrial isolation kit (chemical method) (Pierce Biotechnology Inc., Rockford, IL). To receive the total cellular proteins, cells were harvested and lysed in cold lysis buffer (10% v/v glycerol, 1% v/v Triton X-100, 1 mM sodium orthovanadate, 1 mM EGTA, 10 mM NaF, 1 mM sodium pyrophosphate, 20 mM Tris, pH 7.9, 100 µM β-glycerophosphate, 137 mM NaCl, 5 mM EDTA, 1 mM phenylmethylsulfonylfluoride, 10 µg/ml aprotinin and 10 µg/ml leupeptin), homogenized, centrifuged, and then the supernatant was boiled in loading buffer with an aliquot corresponding to 50–100 µg of protein. Samples were then separated by SDS-PAGE and transferred to PVDF membranes. After blotting, PVDF membranes were incubated with primary antibodies for 2 hours and with the secondary antibody labeled with horseradish-peroxidase for 1 hour. The antigen–antibody complexes were visualized by enhanced chemiluminescence. In some experiments 10 µM Z-DON from Zedira, a cell permeable inhibitor of TG2 (IC 0.02 µM) was added to the cells.

### Immunofluorescence staining and confocal microscopy

Cells were treated with Dox for 18 h and then loaded with 3 µM Rhod-2 AM for another 30 min, as described above. Cells were harvested, deposed onto coverslips, centrifuged at 300 rpm for 10 min and fixed for 5 min in methanol. TG2 immunofluorescence analyses were performed using a monoclonal anti-TG2 antibody (1∶50) and FITC-conjugated anti-mouse IgG secondary Ab. Cells were visualized using an inverted laser-scanning confocal microscope (LSM 410, Zeiss) with an ×63/1.4 oil-immersion objective.

### [Ca^2+^]_mito_ determination with Rhod 2-AM

Cells were cultured in RPMI1640 without phenol red containing 3 µM Rhod2-AM (Invitrogen, Carlsbad, CA, USA) for 30 minutes. They were then washed two times with RPMI without phenol red and resuspended in the same medium. The changes in the fluorescence of the Rhod2-AM dye (λexc = 540 nm;λem = 590 nm) were measured in a microplate reader (MD Flexstation 3). In the case of Dox-treated cells, after the reading the cells were counted with trypan blue to normalize the fluorescence value for living cells. Mitochondrial calcium levels were presented as the ratio of Dox-treated cells versus untreated cells. In case of thapsigargin (5 µM) or ATP (500 µM) treatments basal mitochondrial Ca^2+^ levels are shown as 100%.

### [Ca^2+^]_cyto_ determination with Fura-2

Cytosolic Ca^2+^ level, [Ca^2+^]_cyto_, was measured using Fura-2 (Invitrogen, Carlsbad, CA, USA). Cells were cultured in RPMI1640 without phenol red containing 2 µM Fura 2-AM (Invitrogen, Carlsbad, CA, USA) for 45 minutes. Then, cells were washed two times with RPMI without phenol red and resuspended in the same medium. The variation of fluorescence of the Fura-2 dye (λexc = 340 nm and 380 nm; λem = 510 nm) were measured in microplate reader (MD Flexstation 3). Cytosolic Ca^2+^ levels were presented as the ratio of value in Dox-treated cells versus untreated cells at the starting time point. Cytosolic Ca^2+^ levels are presented as a 340/380 Fura-2 ratio.

### [Ca^2+^]_ER_ determination with Mag-Fura-2 AM

Endoplasmic reticulum calcium level, [Ca^2+^]_ER_, was measured using Mag-Fura-2 (Invitrogen, Carlsbad, CA, USA). Cells were cultured in medium containing 5 µM Mag-Fura 2-AM (Invitrogen, Carlsbad, CA, USA) for 45 minutes. Cells were then washed two times with intracellular calcium buffer (125 mM KCl, 25 mM NaCl, 10 mM HEPES, 0.11 mM CaCl_2_, and 0.1 mM MgCl_2_, pH 7.3) and resuspended in the same medium. To release the dye from the cytoplasm, the cells were switched into intracellular buffer containing 10 µg/ml digitonin so that the only dye remaining was sequestered within membrane-bound organelles, predominantly in the ER. The plasma membranes of cells loaded with Mag-Fura-2 were selectively permeabilized by application of 10 µg/ml digitonin in intracellular buffer containing 0.5 mM EGTA. The changes in the fluorescence of the Mag-Fura-2 dye (λexc = 340 nm and 380 nm; λem = 510 nm) were measured in a microplate reader (MD Flexstation 3). Basal ER calcium levels were measured for 5 min before addition of thapsigargin (5 µM) and are shown as 100%.

### 2-D Gel electrophoresis

Cells were homogenized in lysis buffer (10% v/v glycerol, 1% v/v Triton X-100, 1 mM sodium orthovanadate, 1 mM EGTA, 10 mM NaF, 1 mM sodium pyrophosphate, 20 mM Tris, pH 7.9, 100 µM β-glycerohosphate, 137 mM NaCl, 5 mM EDTA, 1 mM PMSF, 10 µg/ml aprotinin and 10 µg/ml leupeptin). After centrifuging at 14,000 rpm for 15 min to remove cell debris, the supernatant was subjected to 2-D gel electrophoresis. In first dimension, isoelectric focusing (IEF) was performed using the Bio-Rad PROTEAN™ IEF cell (Bio-Rad). 300 µg protein were prepared in 100 µl IEF buffer (8 M urea, 2% CHAPS, 140 mM 2-ME and 0.2% ampholyte) and incubated at 37°C for 30 min. Protein samples were loaded onto a ReadyStripTM IPG Strip (pH 3–10, 7 cm;Bio-Rad) by passive re-hydration for 16 hours. Prior to the second dimension, the focused strips were equilibrated in equilibration buffer I (6 M urea, 0.375 M Tris-HCl, pH 8.8, 2% SDS, 20% glycerol and 2% DTT) for 15 min. Then, the focused strips were equilibrated in equilibration buffer II (6 M urea, 0.375 M Tris-HCl, pH 8.8, 2% SDS, 20% glycerol and 2.5% iodoactamide) for 15 min. Finally, the equilibrated strips were positioned and separated on 12.5% polyacrylamide gel. Protein spots on the gel were detected by Coomassie Brilliant Blue R-250 (CBR) staining. Protein spots analysis and identification were carried out by using LC-MS/MS by the Instrument Center of Chung Shan Medical University.

### Immunoprecipitation of RAP1GDS1

1×10^7^ Tet-On wt TG2 Jurkat cells without treatment, exposed to thapsigargin for 15 min or to doxocycline treatment for 6 hours were harvested, and whole cell lysates were prepared in RIPA buffer containing 50 mM Tris–HCl, pH 8.0, 137 mM NaCl, 10% glycerol, 1% Nonidet P-40 (NP-40), 1 mM sodium vanadate, 10 mM sodium pyrophosphate, 50 mM sodium fluoride, 1 mM phenylmethylsulfonil fluoride, 10 µg/ml leupeptin, and 2 µg/ml aprotinin. Cell lysates were centrifuged, precleared with protein A from Santa Cruz (20 µl) and the isotype control antibody (1 µg), and following centrifugation RAP1GDS1 was immunoprecipitated by anti-RAP1GDS1 antibodies (Antibodies online) and protein A sepharose (Santa Cruz) as it is described in the Santa Cruz immunoprecipitation protocol. Following Western blot analysis immunoprecipitated RAP1GDS1 was tested with both anti-RAP1GDS1 and anti-ε(γ-glutamyl) lysine isopeptide antibodies.

### RNA interference

RNAi was based on lentiviral delivery of shRNA. Lentiviral particles were produced in HEK293T cells by co-transfection of lentiviral vector containing the short hairpin RNA (shRNA) against Rap1GDS1 (Sigma Aldrich) with lentiviral packaging plasmids pMD2G, pRRE and pRSV/REV (Sigma Aldrich) using Lipofectamin2000 (Invitrogen) according to the provider's instructions. Empty vector (Sigma Aldrich) was used as a negative control. Supernatant of the HEK293T cells was harvested at 48 and 54 h after transfection, which was purified by filtration (Millipore) and concentrated on column (Millipore). The virus titer has been determined by HIV-1 p24 Antigen ELISA kit (Zeptometrix).

### Transduction of Jurkat cells

JK-Tet-On wtTG2 cells were plated onto 6 well plates (5×10^4^ cells/well, in serum free RPMI media). Virus containing HEK293T cell supernatants were added to the cells. On the following day cells were cultured in RPMI medium supplemented with 20% FBS. After two days of incubation cells were selected in the presence of 5 µg/ml Puromycin. The amount of Puromycin was determined by kill curve of Jurkat cells. Cells carrying the constructs were validated with Western blot analysis.

### Subcellular Fractionation of JK-Tet-On wtTG2 cells

Subcellular fractionation was performed as previously described [44]. All fractionation steps were carried out at 4°C. Briefly, cells (10^9^) were harvested, washed by centrifugation at 200 *g* for 5 min with PBS, resuspended in homogenization buffer (225 mM mannitol, 75 mM sucrose, 30 mM Tris-HCl, pH 7.4, 0.1 mM EGTA, and 1 mM PMSF) and gently disrupted by Dounce homogenisation. The homogenate was centrifuged twice at 600 *g* for 5 min to remove nuclei and unbroken cells, and then the supernatant was centrifuged at 10,300 *g* for 10 min to pellet crude mitochondria. The resultant supernatant was centrifuged at 15,000 g for 30 min, and then to pellet the ER fraction the supernatant was centrifuged at 100,000 *g* for 90 min (70-Ti rotor, Beckman). The crude mitochondrial fraction, resuspended in isolation buffer (250 mM mannitol, 5 mM HEPES, pH 7.4 and 0.5 mM EGTA), was subjected to Percoll gradient centrifugation (Percoll medium: 225 mM mannitol, 25 mM HEPES pH 7.4, 1 mM EGTA and 30% vol/vol Percoll) in a 10-ml polycarbonate ultracentrifuge tube. After centrifugation at 95,000 *g* for 30 min (SW40 rotor), the mitochondria-associated membrane fraction containing the structural contacts between mitochondria and ER, was retrieved as a diffuse white band located approximately 1/3 down the tube. MAMs were diluted in isolation buffer and centrifuged at 6,300 *g* for 10 min. To pellet the MAMs fraction, the supernatant was centrifuged at 100,000 *g* for 90 min (70-Ti rotor, Beckman). To check the quality of the preparation 10 µg of proteins, quantified using the Bradford assay (Bio-Rad Laboratories), were separated by SDS-PAGE and transferred to PVDF membrane for standard western blotting.We used different markers for the fractions obtained:β-tubulin as a general cytosolic marker, type 3 IP3R (IP3R3) as a marker for ER, Sigma-1 receptor (Sigma-1R) as a MAMs marker, voltage-dependent anion channel (VDAC) as a mitochondrial marker. The close apposition between ER and mitochondrial membranes at MAMs explained the presence of both VDAC in these microdomains.

### Antibodies

Antibodies were purchased from the following sources and used at the indicated dilutions: RAP1GDS1 (1∶500) from Santa Cruz, TG2 (1∶500) from Thermo Scientific, anti-ε(γ-glutamyl) lysine isopeptide (1∶500) from Covalab, β–tubulin (1∶3000), Hsp60 (1∶1000) and Sigma-1R (1∶1000) from Sigma-Aldrich; IP3R3 (1∶300) from BD Biosciences; VDAC (1∶5000) from Abcam; β-actin (1∶1000) from Merck Millipore. Isotype matched, horseradish peroxidase conjugated secondary antibodies (Santa Cruz) were used, followed by detection by chemiluminescence (SuperSignal West Pico Chemiluminscent Substrate).

### Statistical analysis

Statistical analyses for detection of significant differences between the control and experimental groups were carried out by using the one-way analysis of Variance (one-way ANOVA) with the help of Prism 5.0 (GraphPad Software). A *P*-value of <0.05 was considered to be significant.
